# Reviewed article: Micheletti VCD, Rosa RS, Dipp T, Silva LP, Campagnolo PDB, Mercaus LR, Brito JR, Bonin MEM. Cardiovascular risk and statin prescription in primary care: an observational study, São Leopoldo, 2018-2021. Epidemiol Serv Saude. 2025:34;e20240432

**DOI:** 10.1590/S2237-96222025v34e20240432.c

**Published:** 2025-10-20

**Authors:** Sandra Maria do Valle Leone de Oliveira

**Affiliations:** 1 Fundação Oswaldo Cruz, Campo Grande, MS, Brazil

## First round comments

Parabéns pelo estudo, que consideramos um ponto de atenção, para melhorar o monitoramento do risco cardiovascular, que podem ser abordados por meio do manejo laboratorial e tratamento farmacológico. Entre

os pontos de ajuste, destacamos:

1- O título deve ser revisado para incluir o nome do município da Região Metropolitana de Porto Alegre e o período do estudo. A recomendação de evitar siglas e grafar o nome do estado por extenso também deve ser seguida, em consonância com as orientações aos autores. Como possibilidade, Risco Cardiovascular e Prescrição de Estatinas na Atenção Primária de [nome do município], Rio Grande do Sul: Estudo Observacional (2018-2021).

2- Revisar o resumo de forma minuciosa: objetivo ( claro e alinhado, com o último parágrafo na introdução), Revisar a explicação da calculadora HEARTS, com sigla por extenso e o nome atribuído em português. O termo “estudo observacional transversal” é suficiente para descrever o delineamento. A menção ao financiamento não é apropriada nesta seção e deve ser removida. Apresentar as principais análises realizadas, incluindo explicações como “x²(3)”. A conclusão deve estar diretamente ligada aos resultados apresentados no resumo (a conclusão deve responder diretamente ao objetivo do estudo).

3-Atualização dos Dados referentes às doenças cardiovasculares no Brasil, especialmente em relação às

novas evidências disponíveis. Expandir a discussão sobre a relevância da prescrição de estatinas no contexto

do Sistema Único de Saúde (SUS).

4- Descrição das Análises: A seção de métodos deve ser revisada para incluir as principais análises realizadas, com ênfase nas associações estudadas, como o teste de Qui-quadrado, o Teste de Mann-Whitney e as medidas de dispersão. A padronização das medidas, como intervalo de confiança (IC95%), também deve ser ajustada, seguindo o padrão sugerida nas instruções aos autores.

5- Mencionar e discutir a baixa porcentagem de prontuários que continham todas as variáveis necessárias para calcular o risco cardiovascular (47,8%). Isso impacta a completude do estudo e limita sua aplicabilidade. Recomenda-se discutir melhor essa limitação na seção de resultados e nas conclusões.

6- Clareza e Padronização das Tabelas e revisar o uso de Negrito, preferencialmente excluir os negritos.

7-A interpretação dos dados de associação entre fatores de risco e o sexo deve ser mais detalhada, com uma análise mais profunda sobre as diferenças entre os perfis lipídicos e os fatores de risco cardiovascular entre homens e mulheres.

8- É importante mencionar as limitações de maneira clara, como a baixa completude dos dados e possíveis vieses na coleta de informações dos prontuários. Isso tornará o artigo mais robusto e transparente.

Recommendation: Major Revision

## Second round comments

Prezado autor, agradecemos pelos ajustes e atendimento aos pareceristas que tornaram o artigo com uma compreensão fluida e com valor para tomadores de decisão. Entretanto, poucos ajustes, devem ser ajustados:

1- Sugestão:

Incluir a explicação da sigla HEARTS:

“A calculadora HEARTS (Healthy Eating, Active Living, Risk Factors, Treatment, and Systems) é uma ferramenta desenvolvida pela Organização Mundial da Saúde para estratificação do risco cardiovascular.” Mantenha a explicação em português.

2-Explicar o teste de Qui-quadrado:

“Para análise de associações, foi utilizado o Teste de Qui-quadrado (x²(3), onde ‘3’ representa o grau de liberdade).”

3- Retire a frase dos resultados:

Foram coletadas informações de 4601 prontuários dos quais 2199 (47,8%; IC95% 46,4; 49,2) possuíam todas as variáveis necessárias para o cálculo do risco cardiovascular. dos resultados e trabalhe com essa informação nas limitações do estudo. Como por exemplo:

“A baixa completude dos dados (47,8%) limita a generalização dos resultados e sugere a necessidade de melhorias nos registros dos prontuários eletrônicos, que são fundamentais para a tomada de decisão clínica e o planejamento de ações de saúde pública.”

4- Sugestão, revisar frases como:”A maior parcela dos com baixa escolaridade...”Para:”A maioria dos usuários com baixa escolaridade...”

5- Verificar os dados brutos para identificar possíveis erros de registro ou categorias não declaradas (Raça/ cor da pele, escolaridade, histórico de hipertensão e obesidade, faixa de colesterol total e IMC apresentam discrepâncias nos totais).

Recommendation: Minor revision

## Third round comments

O artigo necessita de pequenas revisões.

Foi identificada uma pequena inconsistência nos Resultados, pois o texto afirma que o grupo com risco cardiovascular alto apresentou 92,2% de histórico de hipertensão, mas a Tabela 1 indica 90,3% (IC95%: 87,2-92,8). Recomenda-se a correção desse valor no texto para garantir alinhamento entre os dados apresentados. Faça a revisão ou justifique a sua permanência.

Recommendation: Minor revision

